# Development of Surgically Transplantable Parathyroid Hormone-Releasing Microbeads

**DOI:** 10.3390/biomedicines10020440

**Published:** 2022-02-14

**Authors:** Ha Yeong Kim, Ji Min Seok, Soo Yeon Jung, Min Ji Lee, An Nguyen-Thuy Tran, Seon Ju Yeo, Su A. Park, Han Su Kim

**Affiliations:** 1 Department of Otorhinolaryngology-Head and Neck Surgery, College of Medicine, Ewha Womans University, 1071 Anyangcheon-ro, Seoul 07985, Korea; ha0@ewha.ac.kr (H.Y.K.); mdjungsy@ewha.ac.kr (S.Y.J.); leeminjibme@gmail.com (M.J.L.); antran1023@gmail.com (A.N.-T.T.); 2 Department of Nature-Inspired System and Application, Korea Institute of Machinery and Materials (KIMM), Daejeon 34103, Korea; jimins@kimm.re.kr (J.M.S.); syeo@kimm.re.kr (S.J.Y.); psa@kimm.re.kr (S.A.P.)

**Keywords:** hypoparathyroidism, alginate, microbeads, tonsil-derived mesenchymal stem cells, parathyroid hormone

## Abstract

Hypoparathyroidism is an endocrine disorder that occurs because of the inability to produce parathyroid hormone (PTH) effectively. Previously, we reported the efficacy of tonsil-derived mesenchymal stem cells (TMSCs) differentiated into parathyroid-like cells for the treatment of hypoparathyroidism. Here, we investigated the feasibility of three-dimensional structural microbeads fabricated with TMSCs and alginate, a natural biodegradable polymer, to treat hypoparathyroidism. Alginate microbeads were fabricated by dropping a 2% (*w*/*v*) alginate solution containing TMSCs into a 5% CaCl_2_ solution and then differentiated into parathyroid-like cells using activin A and sonic hedgehog for 7 days. The protein expression of PTH, a specific marker of the parathyroid gland, was significantly higher in differentiated alginate microbeads with TMSCs (Al-dT) compared with in undifferentiated alginate microbeads with TMSCs. For in vivo experiments, we created the hypoparathyroidism animal model by parathyroidectomy (PTX) and implanted alginate microbeads in the dorsal interscapular region. The PTX rats with Al-dT (PTX+Al-dT) showed the highest survival rate and weight change and a gradual increase in serum intact PTH levels. We also detected a higher expression of PTH in retrieved tissues of PTX+Al-dT using immunofluorescence analysis. This study demonstrates that alginate microbeads are potential a new tool as a surgically scalable therapy for treating hypoparathyroidism.

## 1. Introduction

Hypoparathyroidism is an endocrine disorder that occurs because of the inability to produce parathyroid hormone (PTH) effectively and is the most common result of damaged or removed of parathyroid glands during thyroidectomy [1,2]. The loss of PTH leads to an imbalance of calcium homeostasis in the body, leading to hypocalcemia [3]. Several clinical therapies have been used to treat hypoparathyroidism. A common treatment is the daily intake of multiple doses of calcium and vitamin D [4]. However, this method is not suitable for balancing human mineral levels and can cause hypercalciuria in patients with hypoparathyroidism. Synthetic PTH (1–84) approved by the U.S. Food and Drug Administration (FDA) was developed as a PTH replacement for the treatment of hypoparathyroidism, which has several limitations, including the need for daily injections and short half-life (~4 min) [5,6]. Moreover, its use is currently withheld due to the potential risk of osteosarcoma [7].

The ideal PTH replacement therapy for hypoparathyroidism is an autograft of parathyroid glands. Auto-transplantation is an ideal biocompatible treatment that automatically senses and regulates calcium levels in the body; however, it can only be applied when the parathyroid gland is accidentally resected during surgery [8]. According to this notion, we need to develop new treatments that are easy to apply, long-lasting, and safe to use without any complications. Recently, mesenchymal stem cells (MSCs) have been widely used to develop clinical applications in tissue engineering and regenerative medicine. We previously established tonsil-derived MSCs (TMSCs) as a new source of MSCs and investigated their clinical potential [9]. 

TMSCs have a relatively higher proliferation rate compared with other tissue-derived MSCs and have the multipotency to differentiate into several cell types [10,11,12,13]. In particular, we reported that TMSCs differentiated into parathyroid-like cells as functional replacements for parathyroid glands in vitro and in vivo [14]. In this process, various materials were investigated as a source of scaffold for applying differentiated cells to the body [15,16,17], but they had some limitations for their use in clinical applications. For example, Matrigel^®^ (BD Biosciences, San Jose, CA, USA) is rich in components, such as laminin and entactin, which effectively induce attachment and cell proliferation [18]. However, it has not been approved for clinical use in humans because it is a basement membrane derived from Engelbreth–Holm–Swarm mouse tumors [19]. In contrast to Matrigel^®^, plasma gel is an autologous material that does not require FDA approval and can be easily isolated and obtained from blood. Moreover, it has abundant growth factors and extracellular matrix (ECM) [17]. However, plasma gel is difficult to mix with cells to form proper implants. Therefore, we must develop a safe and suitable scaffold to carry cells.

Alginate is the salt of alginic acid extracted from brown algae and is a natural biodegradable polymer approved by the FDA [20]. Alginate can be fabricated into a microsphere using the most common method, wherein an aqueous alginate solution turns into a hydrogel under the influence of an ionic cross-linking agent, such as calcium chloride (CaCl_2_). It has also been reported that alginate has good scaffold-forming properties for delivering drugs or cells and allows the free movement of nutrients, such as oxygen and therapeutic products, in and out of the scaffold [21,22]. Moreover, it can protect against physical stress and maintain viability for long-term cultures [23]. Owing to these properties, encapsulating cells with alginate can be widely used for therapeutic purposes to treat various diseases.

In this study, we investigated whether alginate microbeads, i.e., TMSCs-encapsulated microbeads fabricated using alginate, are effective scaffolds for the treatment of hypoparathyroidism.

## 2. Materials and Methods

### 2.1. Isolation and Differentiation of TMSCs

TMSCs were isolated and expanded as previously described [9]. Briefly, we obtained tonsil tissues from patients (<10 years old) undergoing tonsillectomy with informed written consent. This protocol was approved by the Institutional Review Board (ECT-11-53-02) of Ewha Womans University Mokdong Hospital, Korea. Isolated tonsil tissues were mechanically minced with scissors and then enzymatically digested in Dulbecco’s Modified Eagle’s Medium containing high glucose (4500 mg/L) (DMEM-HG; Welgene Inc., Gyeongsan, Korea) supplemented with 10% fetal bovine serum (FBS; Corning, Corning, NY, USA), 100 U/mL penicillin, 100 U/mL streptomycin, 0.25 μg/mL amphotericin B, 210 U/mL collagenase type I (Thermo Fisher Scientific, Waltham, MA, USA), and 10 mg/mL DNase (Sigma–Aldrich, St. Louis, MO, USA) at 37 °C for 30 min. After filtering the digested tissues, mononuclear cells were obtained by density gradient centrifugation using Ficoll-Paque (GE Healthcare, Piscataway, NJ, USA) and were then plated in a T-150 culture flask for 48 h. 

Nonadherent cells were discarded, and adherent cells were further incubated in the culture medium (DMEM-HG containing 10% FBS, 100 U/mL penicillin, 100 U/mL streptomycin, and 0.25 μg/mL amphotericin B). TMSCs were used between passages 4–7 for all experiments.

### 2.2. Preparation and Differentiation of Alginate Microbeads

We used alginic acid sodium salt (Sigma–Aldrich) to prepare alginate microbeads. The final 2% (*w*/*v*) alginate solution was dissolved in the culture medium and then gently mixed with TMSCs (2 × 10^7^/mL). For encapsulating cells, alginate solution containing TMSCs was extruded into a cross-linking bath of 5% CaCl_2_ solution by a syringe pump (KDS Legato 100, KD Scientific, Holliston, MA, USA) at a flow rate of 0.02 μL/mL. In this process, nozzles of 32, 27, and 22 gauge (G) were used. The prepared alginate microbeads were washed twice with Dulbecco’s phosphate-buffered saline (DPBS; Welgene Inc.) and once with the culture medium.

We used the modified Bingham protocol for differentiating TMSCs into parathyroid-like cells [24]. Briefly, alginate microbeads with TMSCs were placed into 12-well plates with five microbeads for each well and cultured without or with a differentiation medium containing 100 ng/mL recombinant human activin A (R&D Systems, Inc., Minneapolis, MN, USA) and 100 ng/mL recombinant human sonic hedgehog (Shh; R&D Systems) for 7 days; undifferentiated control alginate microbeads with TMSCs (Al-cT) cultured in culture medium; and differentiated alginate microbeads with TMSCs (Al-dT) cultured in differentiation medium. The medium was changed every 3 or 4 days.

### 2.3. Decapsulation of Alginate Microbeads

Alginate microbeads can be decapsulated using ethylenediaminetetraacetic acid (EDTA). For decapsulating, alginate microbeads were washed twice with DPBS, and then single microbeads were transferred to new Eppendorf tubes (E-tube). The decapsulating solution (0.05 M EDTA, pH 8.0) was added to the E-tube containing microbeads and incubated for 30 min at 37 °C in a humidified CO_2_ incubator. After that, the cell pellet was obtained by centrifugation and resuspended. Resuspended cells were counted using a hemocytometer.

### 2.4. Measurement of the Size and Morphology of Alginate Microbeads

The size and morphology of alginate microbeads were observed under an optical microscope (Olympus, BX51, Tokyo, Japan) and analyzed using ImageJ software (National Institutes of Health, Bethesda, MD, USA). 

We also examined the microstructure of alginate microbeads using a scanning electron microscope (SEM; FEI, Hillsboro, OR, USA). For primary fixation, alginate microbeads were fixed with 2.5% glutaraldehyde in 0.1 M phosphate buffer for 1 h and then washed three times with DPBS. Secondary fixation was performed using 1% osmium in 0.1 M phosphate buffer for 1 h, followed by three washes with DPBS. The fixated alginate microbeads were dehydrated with 50%, 70%, 90%, and 100% ethanol for 5 min and dried overnight at room temperature. These specimens were then cut in half, mounted on a coaster, and coated with chromium. The cross-section of the specimen was observed under SEM.

### 2.5. Cell Viability Assay

The cell viability of alginate microbeads was determined using a Live/Dead assay consisting of two reagents (Thermo Fisher Scientific): calcein-acetoxymethyl ester and ethidium homodimer-1. Live/Dead assay reagents were prepared by dilution to a concentration of 50 mM calcein-acetoxymethyl ester and 25 mg/mL ethidium homodimer-1 in the culture medium. Alginate microbeads were incubated with Live/Dead assay reagents for 30 min at 37 °C in a humidified CO_2_ incubator. Fluorescence images were obtained using an inverted fluorescence microscope (Nikon Ti2-U, Nikon, Tokyo, Japan).

### 2.6. In Vivo Animal Experiments and the Development of Hypoparathyroidism Animal Model

The in vivo animal study was approved by the Institutional Animal Care and Use Committee (IACUC) at Ewha Womans University (IACUC Approval No. EUM20-004), Korea. We randomly allocated male Sprague–Dawley rats (Orient Bio, Seongnam, Korea), weighing approximately 300 g, to all the experimental groups. All animals were housed under a 12-h light/dark cycle and provided with an AIN-93G diet (Research Diets, New Brunswick, NJ, USA) that contained 5 g/kg calcium (0.5%) during experimental periods. Except for the sham group, other groups underwent parathyroidectomy (PTX) as described in the previous study [15]. 

To summarize, the sham group underwent a sham operation (*n* = 7), the PTX+Al group underwent PTX and implanted alginate microbeads without cells (*n* = 6), the PTX+Al-cT group underwent PTX and implanted Al-cT (*n* = 8), and the PTX+Al-dT group underwent PTX and implanted Al-dT (*n* = 8). Briefly, we intraperitoneally injected 5-aminolevulinic acid (5-ALA; Sigma–Aldrich) to PTX groups (PTX+Al, PTX+Al-cT, and PTX+Al-dT). After 2 h, animals were anesthetized with Zoletil (Virbac Korea, Seoul, Korea) and xylazine chloride (Bayer Korea, Seoul, Korea; 1:1 mix, 0.1 mL/100 g), and then, the skin of the neck was incised. Trachea and thyroid glands were exposed, and parathyroid glands were detected by red fluorescence under xenon light illumination with an ultraviolet filter (390–440 nm). We removed the parathyroid glands gently and closed the incision with 4-0 Ethilon^®^ sutures (Johnson & Johnson, New Brunswick, NJ, USA). The PTX groups received another longitudinal skin incision in the dorsal interscapular region and were implanted with the corresponding microbeads (five microbeads/rat).

We measured the survival rates and body weight changes of the surviving experimental animals at each time point. The number of surviving animals is shown in Supplementary Table S1.

### 2.7. Assessment of iPTH

We collected animal serum by jugular vein puncture before surgery and at 7, 14, 28, 56, and 84 days after implantation. Intact PTH (iPTH) levels were analyzed using a Rat Bioactive Intact PTH enzyme-linked immunosorbent assay (ELISA) kit (Immutopics, San Clemente, CA, USA) to measure the serum from the sham group and the serum collected before surgery from the PTX groups. In the PTX groups, serum samples collected after implantation of microbeads were analyzed using a human bioactive PTH 1–84 ELISA kit (Immutopics).

### 2.8. Histological Evaluation

Animals were sacrificed 12 weeks after implantation for histological evaluation. Tissues implanted with alginate microbeads were retrieved from all animals and fixed with 4% (*w*/*v*) paraformaldehyde. The fixed tissues were embedded in paraffin blocks and cut into 5-μm thick sections using the microtome. Sectioned tissues were then stained with hematoxylin and eosin (H&E) and mounted. The images were obtained using a slide scanner (Leica Biosystems, Buffalo Grove, IL, USA).

For the immunofluorescence assay, specimens were rehydrated and blocked with 1% bovine serum albumin solution for 1 h. After blocking, the samples were incubated with primary antibodies for the following target proteins: human mitochondria (Sigma–Aldrich), and PTH (Ab Frontier, Seoul, Korea) (each at a 1:100 dilution), followed by the addition of the corresponding Alexa Fluor 488-conjugated anti-mouse or -rabbit secondary antibodies (Thermo Fisher Scientific). Lastly, nuclei were counterstained with 4′,6-diamidino-2-phenylindole (DAPI). The images were examined using a confocal microscope (LSM5 Pascall, Carl ZEISS, Oberkochen, Germany) or an inverted fluorescence microscope (Nikon Ti2-U).

### 2.9. Statistical Analysis

All results are presented as the mean ± standard deviations (S.D.). Statistical significance was determined using Student’s t-test and one-way analysis of variance (ANOVA). A *p*-value of less than 0.05 was considered statistically significant. All experimental trials were repeated at least three times to validate the reproducibility of the outcomes.

## 3. Results

### 3.1. Characterization of Alginate Microbeads

**Figure 1:**
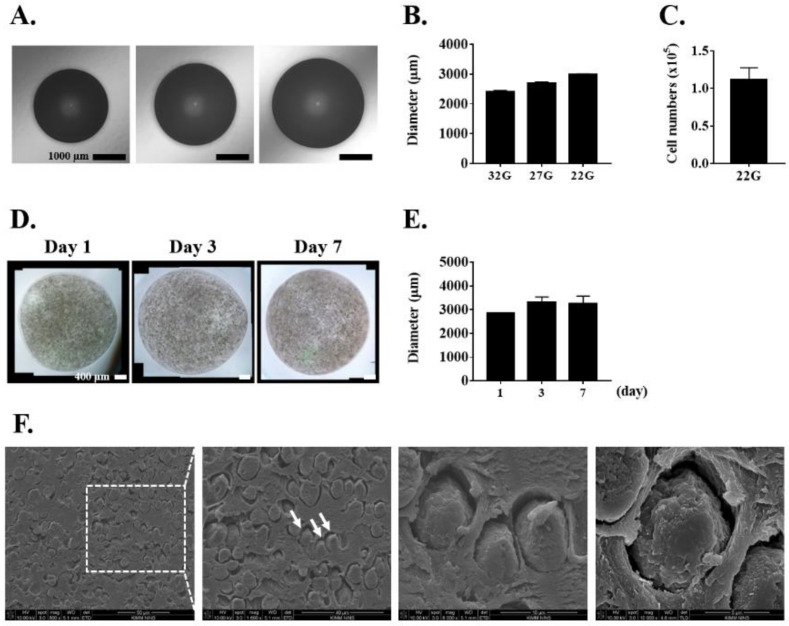
Characterization of alginate microbeads. (**A**) Alginate microbeads fabricated with various nozzle sizes. Scale bar = 1000 μm. (**B**) Measurement of alginate microbeads size. (**C**) The number of cells in alginate microbeads fabricated with a 22 G nozzle. (**D**) The images of alginate microbeads fabricated with a 22 G nozzle at different culture times. Scale bar = 400 μm. (**E**) Measurement of alginate microbeads size at different culture times. (**F**) The encapsulated cells indicated by white arrows were measured using SEM imaging at day 1. Scale bar = 40 μm. Results are representative of at least three independent experimental repeats. Bars indicate mean ± S.D.

We fabricated alginate microbeads using alginic acid sodium salt, wherein the size of the alginate microbeads was controlled using various nozzle sizes. The diameters of microbeads prepared with 32, 27, and 22 G nozzle sizes were 2417.5 ± 39.5, 2693.1 ± 43.0, and 2984.9 ± 26.4 μm, respectively (Figure 1A,B). Larger diameter microbeads were obtained when using a smaller number of nozzles at the same flow rate as that of the syringe pump. Among these various sizes, alginate microbeads made with a 22 G nozzle were suitable due to their feasible size for cell encapsulation. 

We counted the number of cells by decapsulating alginate microbeads and confirmed that a single microbead contained approximately 1.1 × 10^5^ cells/microbead (Figure 1C). After fabrication, the size and structure of the alginate microbeads were well maintained for up to 7 days; the diameters at day 1, 3, and 7 were 2847.0 ± 8.5, 3307.8 ± 225.9, and 3265.8 ± 303.7 μm, respectively (Figure 1D,E). As shown in Figure 1F, SEM images revealed that the encapsulated cells were uniformly distributed inside the beads and that the cell morphology was also well maintained.

### 3.2. Characterization of Alginate Microbeads

**Figure 2:**
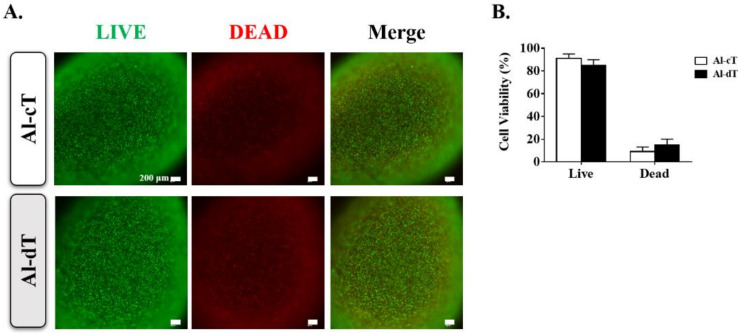
The cell viability analysis of alginate microbeads. The in vitro cell viability was evaluated using a Live/Dead assay. (**A**) Live/Dead assay performed at day 7. Staining of Al-cT and Al-dT for live cells (green) and dead cells (red). (**B**) Quantification analysis of fluorescence intensities. The results are representative of at least three independent experimental repeats. Bars indicate the mean ± S.D.

Previously, we reported that TMSCs were differentiated into parathyroid-like cells with treatment of activin A and Shh [14]. Based on this method, microbeads were exposed to activin A and Shh for 7 days. We examined the cell viability of Al-cT and Al-dT at day 7 using a Live/Dead assay (Figure 2A). As shown in Figure 2B, there was no significant difference in the viability of cells between the two groups, i.e., Al-cT was 90.9 ± 4.0% viable and Al-dT was 85.0 ± 5.0% viable. We also confirmed that the cells were well distributed even after 7 days.

**Figure 3:**
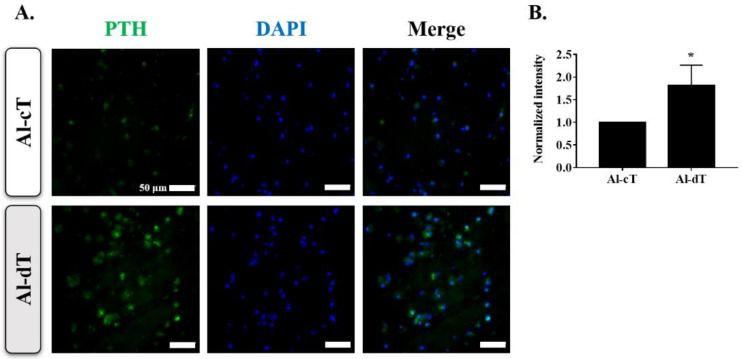
Immunofluorescence analysis of alginate microbeads. (**A**) Representative images of two groups showing PTH (green) and DAPI (blue). (**B**) Quantitative analysis of PTH intensity normalized to nuclear fluorescence. The results are representative of at least three independent experimental repeats. Bars indicate the mean ± S.D. Statistically significant difference is denoted as * *p* < 0.05.

To investigate whether alginate microbeads differentiated into parathyroid-like cells, immunofluorescence analysis was used to examine the protein expression of PTH, a specific marker of the parathyroid gland. As expected, PTH expression was significantly higher in Al-dT than in Al-cT (Figure 3A,B).

### 3.3. The Measurement of Survival Rates and Body Weight Changes

**Figure 4:**
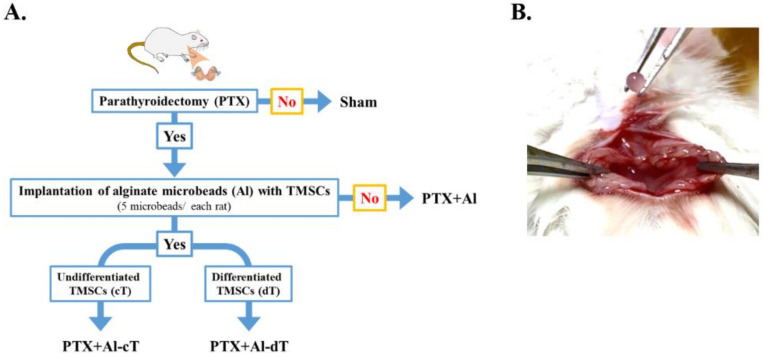
Schematic diagram of in vivo experimental groups (**A**) and image of the implantation procedure (**B**).

**Figure 5:**
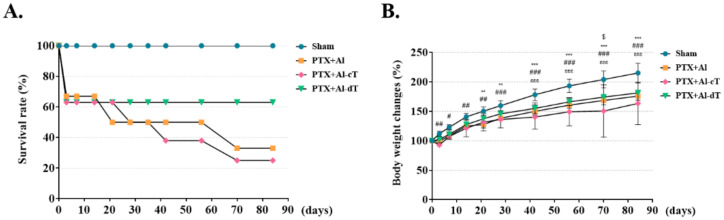
Measurement of survival rates and body weight changes. The in vivo therapeutic efficacy of alginate microbeads was confirmed using the PTX animal model. (**A**) Survival rates and (**B**) body weight changes were measured at various time points during the experiment. Statistically significant differences between Sham and PTX+Al are denoted as ** *p* < 0.01 and *** *p* < 0.001; statistically significant differences between Sham and PTX+Al-cT are denoted as # *p* < 0.05, ## *p* < 0.01, and ### *p* < 0.001; statistically significant difference between Sham and PTX+Al-dT is denoted as εεε *p* < 0.001; statistically significant difference between PTX+Al-cT and PTX+Al-dT is denoted as $ *p* < 0.05.

We next investigated the effects of alginate microbeads in in vivo animal experiments (Figure 4). All of the sham groups survived until the end of the experiment. Excluding the sham group, the survival rate was the highest at 63% in the PTX+Al-dT. The survival rates of PTX+Al and PTX+Al-cT were 33% and 25%, respectively (Figure 5A). The body weights of all experimental groups increased throughout the experimental periods. Among the PTX groups, the body weight changes were highest in the PTX+Al-dT group with 181%, followed by PTX+Al and PTX+Al-cT with 176% and 163%, respectively (Figure 5B).

### 3.4. Assessment of iPTH Levels and Implanted Alginate Microbeads in PTX Rats

**Figure 6:**
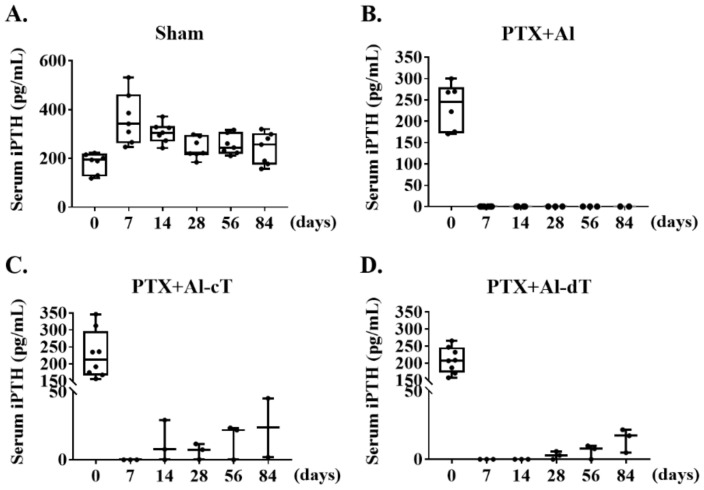
Assessment of the serum iPTH levels in each experimental animal group. (**A**) The sham group showed a normal range of iPTH during the experiment. Except for the sham group, iPTH levels in all experimental groups decreased after PTX. (**B**) Reduced iPTH was detected in PTX+Al until the end of the experiment. (**C**) PTX+Al-cT and (**D**) PTX+Al-dT showed iPTH levels, but PTX+Al-dT only statically restored iPTH.

In the PTX groups, serum iPTH levels decreased to undetectable levels after parathyroidectomy (Figure 6A). In particular, serum iPTH of PTX+Al was not detected until the end of the experiment (Figure 6B). In contrast, we detected the serum iPTH levels in groups implanted with alginate microbeads containing TMSCs (Figure 6C,D), namely, PTX+Al-cT and PTX+Al-dT. Only PTX+Al-dT showed a modest increase in serum iPTH levels after 14 days compared to PTX+Al-cT. However, we observed that the iPTH levels in both groups were not restored to the physiological ranges of serum iPTH.

We also examined serum ionized calcium (iCa^2+^) levels in each group of the experimental animals. Compared to Sham, iCa^2+^ levels were not fully restored to the normal range in all PTX groups, as shown in Figure S1. However, PTX+Al-dT showed a higher mean of serum iCa^2+^ levels than PTX+Al-cT, and maintained constant serum iCa^2+^ levels for 14 to 84 days after surgery.

**Figure 7:**
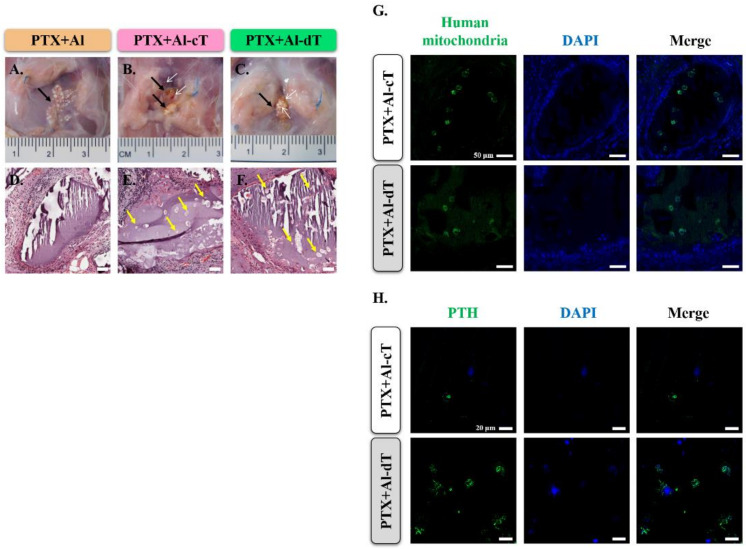
Detection of implanted TMSCs with alginate microbeads. (**A**–**C**) At 12 weeks after implantation, alginate microbeads, indicated by black arrows, were detected in the PTX animal model. The white arrows indicate the formation of the blood vessels. (**D**–**F**) Histological evaluation of implanted alginate microbeads. TMSCs were identified in PTX+Al-cT and PTX+Al-dT (yellow arrows). Scale bar = 50 μm. (**G**) The presence of TMSCs inside alginate microbeads was confirmed by immunofluorescence analysis using human mitochondria antibody (green). Scale bar = 50 μm. (**H**) The expression of PTH in implanted alginate microbeads. Immunofluorescence analysis revealed that the expression of PTH (green) was more extensive in PTX+Al-dT compared to that in PTX+Al-cT. Scale bar = 20 μm.

We evaluated the existence of implanted microbeads in PTX groups and confirmed the implanted microbeads in all PTX rats (Figure 7A–C, black arrows). Furthermore, blood vessels were identified only at the implantation site of alginate microbeads containing TMSCs (Figure 7A–C, white arrows). We also used H&E staining and immunofluorescence analysis to detect TMSCs within the implanted microbeads. As shown in Figure 7D–F, cells were identified in PTX+Al-cT and PTX+Al-dT (yellow arrows), except for PTX+Al. Furthermore, we investigated whether the cells within the implanted microbeads were TMSCs of human tissue origin. As expected, we confirmed the presence of TMSCs in the implanted microbeads in both PTX+Al-cT and PTX+Al-dT by immunofluorescence analysis using human mitochondria antibodies (Figure 7G).

We next examined whether the implanted microbeads expressed PTH protein. There was no expression of PTH protein in PTX+Al (data not shown). Although the expression of PTH was detected in both PTX+Al-cT and PTX+Al-dT, it was higher in PTX+Al-dT (Figure 7H).

## 4. Discussion

Hypoparathyroidism is a medical condition caused by the loss of PTH, and it frequently occurs on damaged or removed parathyroid glands as an accidental excision during thyroid surgeries [1,2]. Several treatments, such as supplementation with calcium and vitamin D or synthetic PTH (1–84) injections, have been used for treating hypoparathyroidism; however, they can cause certain challenges, including secondary side effects or limitations in long-term use [4,5]. To overcome these limitations, researchers have been studying alternative treatments using stem cells in tissue engineering technology. In particular, various scaffolds were applied to load stem cells to prevent their distribution into the body and facilitate their long-term use. In the current study, we found that TMSCs-encapsulated microbeads fabricated using alginate effectively differentiated into parathyroid-like cells by expressing PTH. We also confirmed that implantation of Al-dT increased survival rates and stably restored serum iPTH levels in the PTX animal model. These results may suggest the potential of alginate microbeads fabricated with TMSCs in the treatment of hypoparathyroidism.

In general, cells were maintained in two-dimensional (2D) cell culture systems used to investigate cellular responses to biophysical and biochemical stimuli. Although 2D culture systems allow simple and inexpensive cell culture maintenance and various functional tests, they cannot mimic the natural structure of cells or tissues. In contrast, three-dimensional (3D) cell culture techniques have been developed through various methods for modeling and simulating in vivo conditions [25]. In a previous study, TMSCs were differentiated into PTH-releasing parathyroid cells through 3D spheroidal culture, thus, promising in vivo studies [26]. However, in the case of spheroidal cultured cells, there are some limitations for practical clinical application. As spheroids have a micro size of over ~150 μm, they are too small to be applied in actual clinical practice; therefore, there is a possibility that they will be dispersed in the human body. Furthermore, it was reported that a spheroid larger than ~150 μm in size has a limitation regarding the oxygen/nutrient supply at its core [27]. Therefore, we further explored an ideal cell-carrying system for use in clinical applications and developed a 3D cell-carrying scaffold using hydrogels as an alternative.

Hydrogels are fabricated with different types of biopolymers and are widely used for 3D cell culture because they can easily encapsulate the cells within materials [28]. Alginate, a type of hydrogel, is an FDA-approved natural polymer extracted from brown algae and is safe to use due to its low toxicity, biocompatibility, and biodegradability [29]. Similar to native ECM, alginate mimics natural physical and chemical environments while improving cell functions, such as proliferation, cell viability, and differentiation potentials [30,31]. In this regard, we fabricated cell-carrying beads of various sizes using alginate and confirmed that alginate microbeads retain both structure and encapsulated cells during in vitro and in vivo experiments. We made alginate microbeads using a 2% alginate solution that could produce adequate stiffness and good viability. After 7 days of culture, we observed approximately 80–85% cell viability on the microbeads and found that 10–15% of the cells were dead. This is not high relative to another study where alginate beads made via the same extrusion process died in the range of 15–20% [32]. Nevertheless, in our experiments, the factors affecting cell death may, in part, be the stress generated by air extrusion, which can damage cells. 

In particular, the implanted alginate microbeads remained at the implantation site without dispersal until 12 weeks after implantation and without any signs of inflammation or fibrosis. Moreover, it is interesting to note that more blood vessels were generated within the implanted alginate microbeads with TMSCs than within alginate microbeads without TMSCs in PTX rats (Figure 7A–C, white arrows). Similar to our results, it was also reported that alginate scaffolds combined with MSCs increase vessel formation and tissue-engineered bone regeneration in a nude mouse subcutaneous bone formation model [33]. All these results demonstrate that alginate has a strong advantage as a material for 3D cell cultures and clinical applications.

Previously, studies reported that TMSCs require a 7-day differentiation period to differentiate into PTH-releasing parathyroid cells in 2D or 3D culture systems; the differentiation period of 7 days is sufficient time for both culture systems [14,26]. Consistent with this previous study, we also demonstrated that Al-dT differentiated for 7 days significantly increased the level of PTH compared with Al-cT. Immunofluorescence assays also clearly revealed that PTH was expressed higher in implanted tissues retrieved from PTX+Al-dT than in PTX+Al-cT. 

In addition, there was a gradual increase in serum iPTH levels only in PTX+Al-dT; however, this did not restore normal physiological levels of iPTH. This is because it has been reported that iPTH levels returned close to the normal range when each PTX rat received ~1 × 10^6^ cells/injection [16]. Therefore, we may not have assigned a sufficient number of cells to the PTX rat; we applied approximately ~5.5 × 10^5^ cells per PTX rat. We also detected a wide range of iPTH in PTX+Al-cT, similar to our previous study [3]. It has been reported that the thymus can function as an auxiliary organ expressing and releasing PTH in animals [34,35,36]. However, the PTH secreted by the thymus is uncontrolled and is released more than normal relative to the parathyroid gland [37]. In this context, it is likely that the released PTH in PTX+Al-cT is uncontrolled and that nonfunctional PTH originated from the thymus according to the results, which include the unrecovered survival rates and body weights in Figure 5 [14]. Further studies are needed to clarify how undifferentiated TMSCs control the expression of PTH. Nonetheless, these results suggest that only Al-dT can control and continuously release PTH in PTX rats, thereby, indicating improvements in their survival rate and body weight.

## 5. Conclusions

In the present study, we demonstrated that TMSCs-based microbeads fabricated with a natural compound, alginic acid, are biocompatible, nontoxic, and implantable in the treatment of hypoparathyroidism. Our results suggest that the development of alginate microbeads has the potential to provide clinical applications for parathyroid regeneration.

## Data Availability

Not applicable.

## Supplementary Materials

The following supporting information can be downloaded at: https://www.mdpi.com/article/10.3390/biomedicines10020440/s1, Figure S1: assessment of Serum iCa^2+^ levels in each experimental animal group; Table S1: number of surviving animals.
