# Supplementary material for: Development of Surgically Transplantable Parathyroid Hormone-Releasing Microbeads
Source: Biomedicines. 2022 Feb 14;10(2):440. doi: 10.3390/biomedicines10020440 (PMC8962264; doi:10.3390/biomedicines10020440)
Supplement: Supplementary file 1 [file biomedicines-10-00440-s001.zip › biomedicines-1535214-supplementary.pdf]

Supplementary Materials:

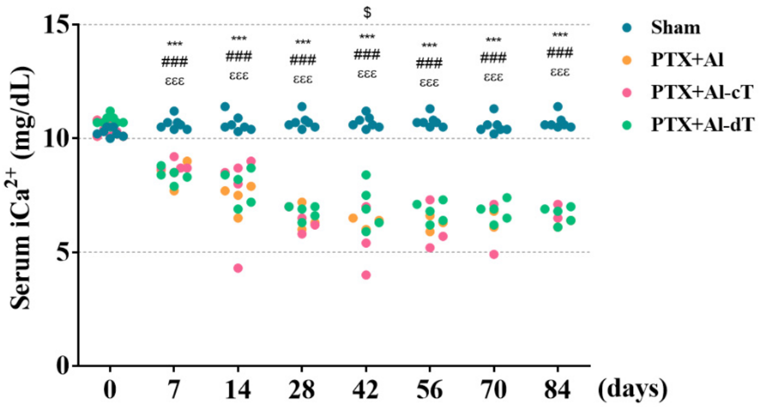

Figure S1. Assessment of Serum iCa^{2+} levels in each experimental animal group. Statistically significant differences between Sham and PTX+Al is denoted as *** $p < 0.001$; statistically significant differences between Sham and PTX+Al-cT is denoted as ### $p < 0.001$; statistically significant difference between Sham and PTX+Al-dT is denoted as εεε $p < 0.001$; statistically significant difference between PTX+Al-cT and PTX+Al-dT is denoted as \$ $p < 0.05$.

Supplementary Table S1. Number of surviving animals

days	Sham	PTX+Al	PTX+Al-cT	PTX+Al-dT
**0**	7	6	8	8
**3**	7	4	5	5
**7**	7	4	5	5
**14**	7	4	5	5
**21**	7	3	5	5
**28**	7	3	4	5
**35**	7	3	4	5
**42**	7	3	3	5
**56**	7	3	3	5
**70**	7	2	2	5
**84**	7	2	2	5
